# Stereotactic IMRT for prostate cancer: Setup accuracy of a new stereotactic body localization system

**DOI:** 10.1120/jacmp.v5i2.1947

**Published:** 2004-08-16

**Authors:** Lu Wang, Rojymon Jacob, Lili Chen, Steve Feigenberg, Andre Konski, Charlie Ma, Benjamin Movsas

**Affiliations:** ^1^ Department of Radiation Oncology Fox Chase Cancer Center Philadelphia Pennsylvania 19111

**Keywords:** body localizer, immobilization, setup accuracy, stereotactic radiotherapy

## Abstract

The purpose of this work is to prospectively assess the setup accuracy that can be achieved with a stereotactic body localizer (SBL) in immobilizing patients for stereotactic intensity‐modulated radiotherapy (IMRT) for prostate cancer. By quantifying this important factor and target mobility in the SBL, we expect to provide a guideline for selecting planning target volume margins for stereotactic treatment planning. We analyzed data from 40 computed tomography (CT) studies (with slice thickness of 3 mm) involving 10 patients with prostate cancer. Each patient had four sets of CT scans during the course of radiotherapy. For the purpose of this study, all four sets of CT scans were obtained with the patients immobilized in a customized body pillow formed by vacuum suction. Unlike other immobilization devices, this system consists not only of a customized body pillow, but also of a fixation sheet used to suppress patient respiratory motion, a stereotactic body frame to provide stereotaxy, and a carbon fiber base board to which both the body cushion and the frame are affixed. We identified four bony landmarks and measured their coordinates in the stereotactic body frame on each set of CT scans. The displacements of the bony landmarks from their corresponding positions on the simulation scan (first CT scan) were analyzed in three dimensions in terms of overall, systematic, and random categories. The initial planned isocenter was also marked on the patients' skin with fiducials for each CT study. The distance from each bony landmark to the fiducial‐based isocenter was measured and compared among the four sets of CT scans. The deviations in distances were also compared to those measured from the landmarks to the stereotactic frame center, in order to determine the effectiveness of the rigid body frame in positioning patients with prostate cancer. Target inter‐fraction motion in this system was also studied for five patients by measuring the deviations in distances from the target geometric center to the bony landmarks. Our results showed that the overall setup accuracy had standard deviations (SDs) of 2.58 mm, 2.41 mm, and 3.51 mm in lateral (LAT), anterior‐posterior (AP), and superior‐inferior (SI) directions, respectively. The random component had SDs of 1.72 mm, 2.06 mm, and 2.79 mm, and the systematic component showed SDs of 0.92 mm, –0.27 mm, and 0.90 mm in these three directions. In terms of three‐dimensional vector, the mean displacement over 116 measurements was 3.0 mm with an SD of 1.29 mm. Compared to the rigid reference, the skin‐mark‐based reference was less reliable for patient repositioning in terms of reproducing known bony landmark positions. The mean target mobility relative to the bony landmarks was 2.22±3.45 mm,0.17±1.11 mm, and 0.11±2.69 mm in the AP, LAT, and SI directions, respectively. In conclusion, the body immobilization system has the ability to immobilize prostate cancer patients with satisfactory setup accuracy for fractionated extracranial stereotactic radiotherapy. A rigid frame system serves as a reliable alignment reference in terms of repositioning patients into the planning position, while skin‐based reference showed larger deviations in repositioning patients.

PACS number: 87.53Ly

## I. INTRODUCTION

Stereotactic radiotherapy has traditionally been limited to intracranial applications because the skull provides a rigid structure for affixation of a stereotactic frame. The advantage of the stereotactic technique is that, by utilizing the stereotaxy of a frame associated with the skull, radiation beams can be recentered on the planned location with great reproducibility. The setup accuracy of less than 2 mm has been achieved for intracranial stereotactic radiotherapy using invasive or noninvasive fixation methods.^(1)^ ^,^ ^(2)^ With such accuracy and combined with conformal and intensity‐modulated techniques, focal high‐dose radiation therapy or radiosurgery has been feasible in the brain. Recently, many studies have shown that some extracranial cancer sites (prostate, lung, and liver metastases) may also benefit from high‐dose radiation therapy.^(^ ^3^ ^–^ ^6^ ^)^ Higher radiation doses require a high accuracy of patient repositioning and a reduction of target movement in order to spare nearby critical organs (e.g., rectum, normal lung, heart, and liver tissue) from high radiation fields. The demand for high accuracy has prompted investigators to develop newer treatment setup procedures and techniques. One of the approaches used to improve the setup accuracy is to employ stereotactic techniques in extracranial radiotherapy. Several noninvasive patient body fixation systems have been developed.^(7)^ ^,^ ^(8)^ Studies on the setup accuracy of these body‐fixation devices for immobilizing patients with solitary lung tumor, liver metastases, and spine stereotactic radiotherapy have been reported in the literature.^(^ ^9^ ^–^ ^13^ ^)^

In an effort to achieve hypofractionation for prostate cancer radiotherapy, a stereotactic approach combined with the intensity‐modulated radiotherapy (IMRT) technique using a micro‐multileaf (4 mm leaf width) for prostate cancer patients has been investigated. In our approach, we employed a new body localizer (BL) system that was originally developed at Medical Intelligence Inc. (Schwabmünchen, Germany) and was later modified and integrated by Radionics Inc. (a division of Tyco Healthcare LP, Burlington, MA) with their treatment‐planning system (TPS) (XKnifeRT). This TPS is specifically designed for both intra‐ and extra‐cranial stereotactic conformal radiotherapy and IMRT. As the first step toward stereotactic IMRT for prostate cancer, we have evaluated the accuracy of the BL in patient repositioning.

It should be noted that the BL system (described below) is not only meant to be an immobilization device, but is also a philosophy for treatment application: the stereotactic system of coordinates is used as a definite reference system for target localization and setup instead of anatomical landmarks such as bony structures or skin markers. Because of its twofold function, it is important to assess which type of reference point is better in reproducing patient position: a system based on a rigid frame or a point defined by skin marks. Moreover, since target relocation is a key issue in fractionated treatment, we have studied the mobility (or inter‐fraction motion) of the target in the BL system. Our objectives of this study were twofold: (1) to evaluate the effectiveness of the body localization system in terms of reproducibility of patient position and (2) to provide guidance for selecting a proper margin for the planning target volume associated with prostate IMRT by quantifying target mobility.

## II. METHODS AND MATERIALS

For this study, a clinical protocol was submitted to and approved by the Institutional Review Board for prostate cancer patients to undergo four sets of CT studies during the course of radiotherapy, in addition to the initial CT scan for treatment planning. The additional four sets of CT scans were obtained with the patients immobilized in a customized body pillow formed by vacuum suction. The actual treatment of the patient was still administrated in the alpha cradle and was not altered by their inclusion in this study. Fourteen patients enrolled in this study; two patients dropped from this study during the course of their treatment for personal reasons. Among the 12 patients who completed all four sets of CT scan, 2 had inconsistent setup procedures. Thus, a total of 40 CT studies from 10 patients was available for data analysis.

### A. The body localizer

**Figure 1:**
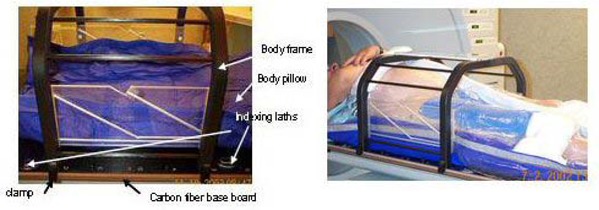
The components of the body localizer system. (a) The base board, body frame, and body pillow; (b) patient immobilized in the body localizer.

**Figure 2:**
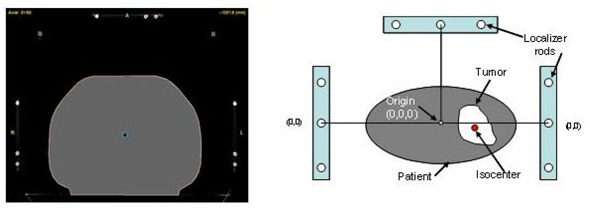
The body localizer consisting of nine rods shown on one slice of the CT image and the coordinate system established by the rods.

The body localizer consists of five components: a whole body pillow made of a large number of small beads of expanded polystyrene, a vacuum system, a fixation sheet, a carbon fiber frame, and a carbon fiber base plate that holds the body cushion and the frame with indexed clamps [Fig. 1(a)]. Fig. 1(b) shows a patient immobilized in this system. The whole body pillow has two indexing bars that can be locked into the base board positioning pins. When it is evacuated, it could maintain the patient's body contour and become rigid enough to support the patient. The vacuum system was used to evacuate air both in the body pillow and between the patient's body and fixation sheet. The fixation sheet was designed to provide pressure, when evacuated, on the patient's body and thus reduce respiration‐related organ motion and other patient movements. The frame consists of nine rods, which define the position of the anatomy within a CT slice relative to the frame center, thus providing the coordinate system for the CT‐scans. Fig. 2 shows one CT image with all nine rods visible and the coordinate system established by these rods.

### B. Setup procedures for CT simulation

During the initial CT simulation, a customized alpha cradle (AC) was first made for each patient while the patient was lying comfortably in the supine position. The inferior edge of the AC was marked on the patient's leg for patient positioning. After positioning the patient in the alpha cradle on the CT couch, a pilot scan was taken to locate the isocenter position, represented by three radio‐opaque marks (1 mm diameter) placed anteriorly at the bottom of the pubic symphysis and two laterally at the middle of the femoral heads. Once the isocenter was defined, the patient was tattooed with a marker. For simulation in the body localizer, an individually shaped body pillow was created during the same session. The patient was placed on the top of the body pillow while it was still inflated with air so that the Styrofoam balls inside the pillow could be easily moved around to conform to the patient's anatomy. The body pillow was fixed to the base board with its indexing bars placed into the pins and had sufficient length extending from the bottom of the patient's feet to the patient's navel. Before the vacuum was applied, the stereotactic frame was rigidly mounted on the base board and centered in the pelvic area. The fixation sheet was used to cover the patient and was firmly pressed to the adhesive tape that surrounded the body pillow. While the vacuum was applied, the patient was brought into the desired position, and the body pillow was modulated to allow for fixation of the body pillow to the stereotactic frame. When the area surrounding the patient's body was air sealed, the fixation sheet pressed against the patient and gently pushed the patient into the body pillow. Once the body pillow became rigid, the vacuum pump was turned off and the customized body cast was completed.

For future patient setup and the repeated CT studies, we recorded the frame position in relation to the base board as indicated by the indexed holes to which the frame was clipped. The position of the cross hair on the frame in the CT gantry reference was also recorded. We also marked the positions of fiducial tattoos on the body pillow for patient longitudinal alignment with respect to the body pillow and the frame. The patient was brought back approximately every 1 to 2 weeks to undergo a total of three more sets of CT scan in the treatment position using the BL system. The radio‐opaque marks (BBs) were replaced on the fiducial tattoos to identify the initial isocenter position. To set up the patient in the BL, we first placed indexing bars of the pillow in the same pin positions on the base board. Then the patient was brought into the body pillow (with the BBs still on the patient skin) and repositioned by adjusting the positions to the best fit into the body cast, by aligning the lateral tattoo positions with the marks on the body pillow. The stereotactic frame was placed at the recorded index position. While no effort was made to reposition the cross‐hair of the frame and the BBs into the original position with respect to the CT gantry reference, their positions in the CT gantry reference were recorded respectively at each session.

A Picker 5000 CT scanner was used for the studies. A spiral CT scan with 3 mm slice thickness and 480 mm field of view was performed (pixel size: 0.9375 mm×0.9375 mm×0.9375 mm), which included the localization system. All patients were instructed to have a full bladder for CT scanning.

### C. Assessments of setup accuracy

Bony landmarks were used for patient position verification based on the assumption that bony landmarks were not mobile within the patient and therefore would not alter the accuracy of patient repositioning. Assessment of the setup accuracy was performed based on the changes of the coordinates of several identified bony landmarks on the treatment CT scans versus those measured on the reference CT scan. A commercial treatment planning system (XKnifeRT2, Radionics Inc., Burlington, MA) was used for this study, since it has the ability to incorporate the three‐dimensional coordinate system provided by the stereotactic frame onto the CT images. We identified four bony landmarks on each set of the CT images displayed with a zoom scale of approximately 2 on the computer screen. The window width and level were kept about the same to ensure the same image contrast for identifying the same positions on the landmarks. The coordinates [e.g., the distances to the frame center in anterior‐posterior (AP) (*y*), lateral (LAT) (*x*), and superior‐inferior (SI) (*z*) directions] of the landmarks were compared between the treatment CT scans (i.e., scans taken after the initial scan) and the reference CT (the initial scan) scan to determine the displacements of the positions of the landmarks in relation to the frame center. For each landmark, three displacements were obtained along each axis by subtracting the coordinate of CT scans #2, #3, and #4 from that of CT scan #1, respectively, and 12 displacements in total were measured for four landmarks for each patient. Except for the first patient who had two reference scans because the body pillow changed between the first and the second CT scans, a total of 116 measurements of the displacement in LAT, AP, and SI directions, respectively, was used for setup accuracy analysis. A negative sign in the AP, LAT, and SI directions means that the bony landmark was shifted toward the posterior, left, and inferior directions, respectively.

The accuracy for identifying the same landmark was studied in terms of the uncertainties in the measurements between different investigators (inter‐investigator uncertainty) and within one investigator (intra‐investigator uncertainty). Both uncertainties were small (within 1 mm) in the *x* and *y* directions. Due to the slice thickness of 3 mm, the measurement along the longitudinal direction had ±1.5 mm uncertainty.

Displacements were studied in three major directions in terms of overall, systematic and random categories based on the model used by Willner et al.^(14)^ The overall setup errors were calculated using all 116 displacement measurements along a specific direction with standard deviations (SDs). The systematic category indicates displacements that were persistent during the entire course of treatment. For individual patients it was represented by the average value of the displacements along a specific coordinate. For the whole group, there was a distribution of systematic errors that was determined by the SD of the values of the average displacement of individual patients. The random error is the SD around each patient's average displacement. For the whole group, the random error was obtained from the individual displacement values of all patients after subtraction of their corresponding means and calculating the SD of the remaining values.

From the displacements in the three major directions (LAT, AP, and SI), we calculated three‐dimensional (3D) displacement vectors by taking the square root of the square summation of the displacements in the LAT, AP, and SI directions. For this quantity, we only calculated the overall mean and its standard deviation.

### D. Assessment of reference reliability

To contrast the reliability of a definitive reference based on a rigid frame from a traditional skin mark‐based reference, we compared the changes of the coordinates of the bony landmarks in relation to the frame and BB‐based reference. This was done on the same sets of the CT images in which the frame and BBs coexisted. Since in each set of CT images the position of a chosen bony landmark was unique in the reference of the CT system, the variations of the displacement, denoted by the SD, of the bony landmark measured in skin –mark‐based reference systems compared to those measured in a frame based reference system could be used to contrast the reliability of the reference system.

During each setup process, the coordinates of both the BL frame center (represented by cross‐hairs on anterior and lateral plates) and the initial isocenter position (denoting the skin mark‐based reference) represented by the patient skin tattoos in the CT reference coordinate system were recorded. The distances from the bony landmarks to the two reference points were measured respectively along the AP, LAT, and SI directions. The displacements of the landmarks relative to the two reference points were determined respectively by comparing the positions on the treatment CT scans to those on the reference CT scan. There were three displacements for each landmark, and a total of nine values was obtained for three landmarks for each patient. We calculated the average displacement (systematic error) out of the nine values and the SD (random error) around each patient's average. The distribution of the systematic error for the whole group was represented by another SD, which was obtained based on the systematic errors of the 10 patients. It should be noted that this SD is reference system‐dependent. The SD of the group represents the distribution of the systematic errors associated with the type of reference system used; in our case, it was either the stereotactic body localizer (SBL) or the skin‐based reference system. We compared the SDs determined by the two reference systems that coexisted in the CT images in this report.

### E. Prostate interfraction mobility in the BL system

We studied the mobility of the target in the BL system in order to provide a guideline for selecting planning target volume margins for stereotactic IMRT for prostate cancer. We calculated the distances from the geometric center of the prostate, the gross tumor volume (GTV), to a bony landmark (i.e., pubic symphysis where the ischial bone is just disconnected from the symphysis on the CT axial image) and compared the displacements of the distances between the treatment CT scans to that of the reference CT scan. The geometric center of the GTV was automatically located by the planning software (XKnifeRT2) after the GTV was segmented. Gross tumor volumes were contoured for each patient in the four sets of CT studies by the same physician. A total of 15 displacements of the geometric center relative to the bony landmark involving five patients was analyzed.

## III. RESULTS

### A. Setup accuracy of the BL

**Figure 3:**
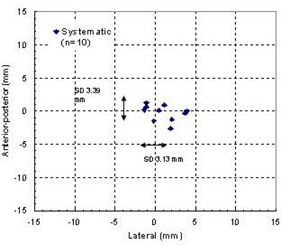
Systematic errors and the range of random errors for individual patients.

Fig. 3 shows the two‐dimensional scatters of the systematic errors obtained for individual patients using 12 displacement measurements per patient (except for patient 1, who had 8 measurements) along the LAT, AP, and SI directions. The random errors for all patients ranged from 0.9 mm to 3.13 mm in the LAT direction, from 0.87 mm to 3.39 mm in the AP direction, and from 1.1 mm to 4.78 mm in the SI direction. Only the maximum SDs are shown in the figure to indicate the maximum range of the random error. The mean setup errors averaged over all 116 displacement measurements and the distributions of systematic and random errors for the whole group are shown in the Table 1. The mean setup error averaged over 116 measurements was 0.96 mm, –0.22, and 0.86 mm in the LAT, AP, and SI directions, respectively. The overall setup error had SDs of 2.58, 2.41, and 3.51 mm along the LAT, AP, and SI axes, respectively. The systematic errors for the group (calculated for 10 patients) had SDs of 1.95, 1.20, and 2.12 mm along these three directions. The random errors for the whole group (calculated for 116 measurements) presented the SDs of 1.72, 2.06, and 2.79 mm in LAT, AP, and SI directions.

**Table 1:** Average displacement and standard deviation (SD) of the overall distribution and the distribution of systematic and random displacements along the three main axes for the whole group

		Distribution of displacements (1 SD (mm))		
Direction	Average displacement (mm) (*n* = 116)	Overall (*n* = 116)	Systematic (*n* = 10)	Random (*n* = 116)
lateral	0.96	2.58	1.95	1.72
anterior‐posterior	–0.22	2.41	1.20	2.06
superior‐inferior	0.86	3.51	2.12	2.79

**Figure 4:**
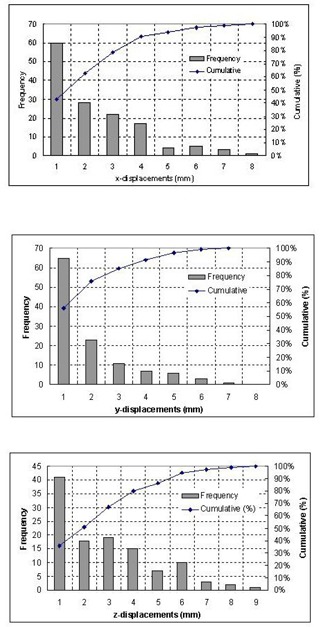
Frequency and cumulative probability of the displacements along the LAT (*x*), AP (*y*), and SI (*z*) directions for total of 116 measurements.

Fig. 4 illustrates the distributions of 116 absolute displacements (no signs were considered) along the three directions. The frequency associated with each range of displacement (in an interval of 1 mm) and the cumulative distribution are presented in this figure. Ninety percent of the setup errors were smaller than 4.5 mm in both the LAT and the AP directions, while in the SI direction, 80% of the setup errors were within 4.5 mm. The setup error was larger in the SI direction due to the slice thickness of 3 mm, resulting in larger uncertainty in the measurement of the landmarks.

**Figure 5:**
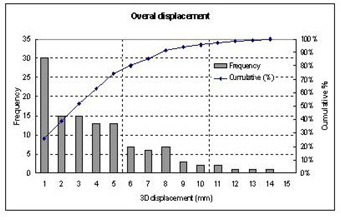
Cumulative distribution of three‐dimensional vector for overall displacement, calculated from 116 sets of displacements for 10 patients.

The cumulative distribution of 3D displacement vectors is shown in the Fig. 5. The overall mean displacement averaged over all 116 displacement vectors was 3 mm with an SD of 1.29 mm. Ninety percent of the 3D displacements were within 7.7 mm. Fig. 5 also shows that the most frequent 3D displacements were in the range of 1–5 mm.

### B. Which reference frame is more reliable?

Table 2 compares the average displacements determined with respect to the two different references over 116 measurements and the distributions of systematic errors (represented by the SDs). By using the frame reference, the systematic components presented SDs of 1.95, 1.2, and 2.12 mm in the LAT, AP, and SI directions, respectively. However, using the skin mark‐based reference, the distributions of the systematic errors represented by the SDs were increased to 3.47, 5.11, and 3.15 mm in these directions. Table 2 also lists the maximum displacements of the identified bony landmarks relative to each reference system. It is seen that the skin –mark‐based reference exhibits the largest maximum displacements. Similarly, the SDs for the systematic errors are larger with the skin‐mark‐based reference compared to those of the frame based reference.

**Table 2:** Comparisons of patient positioning accuracy determined with respect to two different references (the SBL and the BB‐based) in terms of average displacements and the distribution of systematic errors in the three major directions

	Average displacement (mm) (*n* = 116)		1 SD for systematic error (mm) (*n* = 10)		Maximum displacements (mm)	
Directions	Relative to BL	Relative to BB	Relative to BL	Relative to BB	Relative to BL	Relative to BB
left‐right	0.92	–1.41	1.95	3.47	6.8	15.0
anterior‐posterior	–0.27	–0.42	1.20	5.11	7.3	13.55
superior‐inferior	0.90	–0.83	2.12	3.15	9.9	11.3

### C. Target mobility in the BL

**Figure 6:**
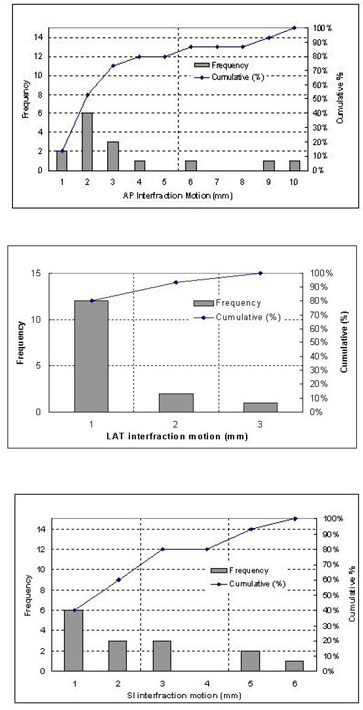
Frequencies and cumulative distributions of the target motion in respect to the patient skeleton along AP, LAT, and SI directions.

Fig. 6 displays the distributions of 15 absolute target displacements (no signs were considered) with respect to the patient skeleton along the AP, LAT, and SI directions. The frequency associated with each range of displacement (in an interval of 1 mm) and the cumulative distribution are presented in this figure. Ninety percent of the target motion was less than 8.5 mm in the AP direction, 2 mm in the LAT direction, and 5 mm in the SI direction. The average displacements (over 15 measurements) of the geometrical center of the GTV relative to the bony landmark were 2.22 mm, 0.17 mm, and 0.11mm in the AP, LAT, and SI directions, respectively. The maximal motion occurred in the AP direction, from 9.6 mm anteriorly to 2.6 mm posteriorly. The second dominant direction of interfraction prostate motion was in SI direction, ranging from 5.7 mm superiorly to 4.5 mm inferiorly.

### IV. DISCUSSION AND CONCLUSION

Unlike the situation in intracranial stereotactic radiosurgery in which the stereotactic frame is affixed rigidly to the patient's anatomy, the localization devices for extracranial stereotactic irradiation are usually noninvasive and thus cannot be rigidly fixed to the patient's anatomy.

Therefore, patient movement in the immobilization device and repositioning uncertainty during a fractionated treatment must be addressed. In this report, we presented the results of our study on the setup accuracy of the body localizer and target interfraction mobility when patients are immobilized in the BL. Our results indicate that, although individual displacement could be as large as 7 mm in the LAT (2 out of 116 measurements) and AP (1 out of 116 measurements) directions and 9 mm (1 out of 116 measurements) in the SI direction, the distributions of the systematic error showed SDs of 1.95 mm in LAT, 1.25 mm in AP, and 2.12 mm in SI directions with the body localizer. A combined 3D setup accuracy overall was 3 mm±1.29 mm. These results are in line with other studies using other body frames. Lohr et al.^(10)^ evaluated a combination of a body cast, hard mask system, and a stereotactic body frame. They reported mean patient movements of 1.6 mm±1.2 mm in the LAT direction, 1.4 mm±1.0 mm in the AP direction, and 2.3 mm±−1.3 mm in the SI direction. Another study by Herfarth et al.^(12)^ presented similar results. The study by Wulf et al.^(11)^ on the treatment accuracy using another stereotactic body frame showed SDs of 3.5 mm in the longitudinal (SI), 2.2 mm in the AP, and 3.9 mm in the LAT directions. This result was obtained from 32 patients treated for primary or metastatic lung cancer, liver metastases, pelvic tumor recurrences, or bone metastases.

Compared to setup uncertainty, target interfraction motion is a major factor affecting treatment uncertainty. Our study showed a range of movement from 9.6 mm anteriorly to 2.6 mm posteriorly and from 5.7 mm superiorly to 4.5 mm inferiorly. The magnitudes of the target interfraction motion were found to be similar to those published results^(^ ^15^ ^–^ ^18^ ^)^ using other immobilization devices. This is understandable since prostate interfraction motion mainly depends on rectal and bladder fillings; the type of immobilization device has little effect on the prostate interfraction motion. However, with the improved setup accuracy, the treatment margin that accounts for both setup accuracy and target interfraction motion can be expected to decrease by 1 to 2 mm for conventional dose fraction scheme. This decrease in margin can reduce the toxicity to the rectum, as shown from several studies.^(^ ^19^ ^–^ ^21^ ^)^ For dose hypofractionation scheme, other methods to reduce the effect of interfraction motion have to be developed. One such approach could be the use of real‐time images, such as CT in the treatment room (i.e., CT‐on‐rails) or electronic portal imaging devices (EPID), to correct the interfraction prostate motion.

Although rigid affixation is difficult for the pelvic region because organs are mobile within the bony structures, the body localizer has a unique application, providing reliable stereotaxy for target reposition using imaging techniques. This feature distinguishes the BL from other immobilization devices. Our underlining assumptions for using the stereotactic approach are as follows: First, with a rigid frame attached to the patient body, the position of any volume within the frame can be defined and repositioned according to the coordinate displacement. Second, the frame center in the treatment machine reference can be relocated; thus radiation can be re‐aimed to the same location. Conventional treatment setups attempt to recreate a particular patient orientation on the treatment couch that is identical to that planned from a CT scan, normally using only a few coplanar reference tattoo markers on the skin. Over the course of treatment, a patient's body surface undergoes changes over time. If the patient's body surface varies in shape, then it follows that the position of any external reference skin markers will also vary, leading to setup errors. In our study, such inherent shortcomings were contrasted when a rigid frame coexisted with the skin –mark‐based reference. Our results showed that a definitive reference based on a rigid frame is superior to the traditionally defined skin mark‐based reference.

Finally, our setup inaccuracy was largest in the SI direction, with only 80% of the setup errors within 4.5 mm. One reason for this result is the CT slice thickness of 3 mm, resulting in larger uncertainties in the measurement of the landmarks. We now recommend using 2 mm slice thickness for CT scan. Another reason is the patient alignment with the body frame. Our experience with the BL showed that special care must be taken to ensure that the patient is properly aligned with respect to the body frame. Some bony landmarks (i.e., ankle, knee, or femur head) are useful for the SI alignment. Other factors that need to be standardized are the vacuum pressure needed to harden the body pillow in order to maintain the patient contour, as well as maintenance of the customized body pillow to avoid leakage.

In conclusion, the stereotactic body immobilization system (BL) has the ability to immobilize prostate patients with satisfactory setup accuracy for fractionated stereotactic conformal treatment and IMRT. Both random and systematic errors in the three major directions are in the range of ±3 mm. Using the body frame as a reference for aligning a patient is advantageous over that of the fiducial skin marks in terms of relocalization of bony landmarks. A rigid frame system serves as a reliable alignment reference for repositioning patients into the planning position. A rigid reference may also facilitate the correction for target inter‐fraction motion using imaging techniques, such as CT‐on‐rails or EPID.

## References

[1] L.J. Verhey , Immobilizing and Positioning Patients for Radiotherapy, Semin. Radiat. Oncol. 5, 100–14 (1995). 10717133 10.1054/SRAO00500100

[2] A. Pirzkall , J. Debus , F. Lohr , M. Fuss , B. Rhein , R. Engenhart–Cabillic , and M. Wannenmacher , Radiosurgery alone or in combination with whole‐brain radiotherapy for brain metastases, J. Clin. Oncol. 16, 3563–9 (1998). 9817276 10.1200/JCO.1998.16.11.3563

[3] G.E. Hanks , K.L. Martz , and J.J. Diamond , The effect of dose on local control of prostate cancer, Int. J. Radiat. Oncol. Biol. Phys. 15, 1299–305 (1988). 3198435 10.1016/0360-3016(88)90224-6

[4] C.A. Perez , M. Bauer , S. Edelstein , B.W. Gillespie , and R. Birch , Impact of tumor control on survival in carcinoma of the lung treated with irradiation, Int. J. Radiat. Oncol. Biol. Phys. 12, 539–47 (1986). 3009368 10.1016/0360-3016(86)90061-1

[5] M.J. Zelefsky , S.A. Leibel , P.B. Gaudin , G.J. Kutcher , N.E. Fleshner , E.S. Venkatramen et al., Dose escalation with three‐dimensional conformal radiation therapy affects the outcome in prostate cancer, Int. J. Radiat. Oncol. Biol. Phys. 41, 491–500 (1998). 9635694 10.1016/s0360-3016(98)00091-1

[6] A. Pollack , G.K. Zagars , G. Starkschall , J.A. Antolak , J.J. Lee , E. Huang et al., Prostate cancer radiation dose response: results of the M. D. Anderson phase III randomized trial, Int. J. Radiat. Oncol. Biol. Phys. 53, 1097–105 (2002). 12128107 10.1016/s0360-3016(02)02829-8

[7] I. Lax , H. Blomgren , I. Naslund , and R. Svanstrom , Stereotactic radiotherapy of malignancies in the abdomen. Methodological aspects, Acta Oncol. 33, 677–83 (1994). 7946448 10.3109/02841869409121782

[8] J. Debus , R. Engenhart–Cabillic , M.V. Knopp , L.R. Schad , W. Schlegel , and M. Wannenmacher , [Image‐oriented planning of minimally invasive conformal irradiation of the head‐neck area], Radiologe 36, 732–6 (1996). 8999450 10.1007/s001170050135

[9] Y. Negoro , Y. Nagata , T. Aoki , T. Mizowaki , N. Araki , K. Takayama et al., The effectiveness of an immobilization device in conformal radiotherapy for lung tumor: reduction of respiratory tumor movement and evaluation of the daily setup accuracy, Int. J. Radiat. Oncol. Biol. Phys. 50, 889–98 (2001). 11429216 10.1016/s0360-3016(01)01516-4

[10] F. Lohr , J. Debus , C. Frank , K. Herfarth , O. Pastyr , B. Rhein et al., Noninvasive patient fixation for extracranial stereotactic radiotherapy, Int. J. Radiat. Oncol. Biol. Phys. 45, 521–7 (1999). 10487580 10.1016/s0360-3016(99)00190-x

[11] J. Wulf , U. Hadinger , U. Oppitz , B. Olshausen , and M. Flentje , Stereotactic radiotherapy of extracranial targets: CT‐simulation and accuracy of treatment in the stereotactic body frame, Radiother. Oncol. 57, 225–36 (2000). 11054527 10.1016/s0167-8140(00)00226-7

[12] K.K. Herfarth , J. Debus , F. Lohr , M.L. Bahner , P. Fritz , A. Hoss et al., Extracranial stereotactic radiation therapy: set‐up accuracy of patients treated for liver metastases, Int. J. Radiat. Oncol. Biol. Phys. 46, 329–35 (2000). 10661339 10.1016/s0360-3016(99)00413-7

[13] A.S. Shiu , E.L. Chang , J.S. Ye , M. Lii , L.D. Rhines , E. Mendel et al., Near simultaneous computed tomography image‐guided stereotactic spinal radiotherapy: An emerging paradigm for achieving true stereotaxy, Int. J. Radiat. Oncol. Biol. Phys. 57, 605–13 (2003). 14529763 10.1016/s0360-3016(03)00792-2

[14] J. Willner , U. Hadinger , M. Neumann , F.J. Schwab , K. Bratengeier , and M. Flentje , Three dimensional variability in patient positioning using bite block immobilization in 3D‐conformal radiation treatment for ENT‐tumors, Radiother. Oncol. 43, 315–21 (1997). 9215794 10.1016/s0167-8140(97)00055-8

[15] J. Wu , T. Haycocks , H. Alasti , G. Ottewell , N. Middlemiss , M. Abdolell et al., Positioning errors and prostate motion during conformal prostate radiotherapy using on‐line isocentre set‐up verification and implanted prostate markers, Radiother. Oncol. 61, 127–33 (2001). 11690677 10.1016/s0167-8140(01)00452-2

[16] A.J. Nederveen , H. Dehnad , U.A. van der Heide , R.J. van Moorselaar , P. Hofman , and J.J. Lagendijk , Comparison of megavoltage position verification for prostate irradiation based on bony anatomy and implanted fiducials, Radiother. Oncol. 68, 81–8 (2003). 12885456 10.1016/s0167-8140(03)00129-4

[17] D.J. Little , L. Dong , L.B. Levy , A. Chandra , and D.A. Kuban , Use of portal images and BAT ultrasonography to measure setup error and organ motion for prostate IMRT: implications for treatment margins, Int. J. Radiat. Oncol. Biol. Phys. 56, 1218–24 (2003). 12873664 10.1016/s0360-3016(03)00290-6

[18] E. Huang , L. Dong , A. Chandra , D.A. Kuban , I.I. Rosen , A. Evans et al., Intrafraction prostate motion during IMRT for prostate cancer, Int. J. Radiat. Oncol. Biol. Phys. 53, 261–8 (2002). 12023128 10.1016/s0360-3016(02)02738-4

[19] W.R. Lee , G.E. Hanks , A.L. Hanlon , T.E. Schultheiss , and M.A. Hunt , Lateral rectal shielding reduces late rectal morbidity following high dose three‐dimensional conformal radiation therapy for clinically localized prostate cancer: Further evidence for a significant dose effect, Int. J. Radiat. Oncol. Biol. Phys. 35, 251–7 (1996). 8635930 10.1016/0360-3016(96)00064-8

[20] M.L. Dirkx , B.J. Heijmen , G.A. Korevaar , M.J. van Os , J.C. Stroom , P.C. Koper et al. Field margin reduction using intensity‐modulated x‐ray beams formed with a multileaf collimator, Int. J. Radiat. Oncol. Biol. Phys. 38, 1123–9 (1997). 9276380 10.1016/s0360-3016(97)00287-3

[21] A. Jackson , Partial irradiation of the rectum, Semin. Radiat. Oncol. 11, 215–23 (2001). 11447578 10.1053/srao.2001.23481

